# Appraisal of biohydrogenating probiotic enzymes for the synthesis of conjugated linoleic acid isomers

**DOI:** 10.3389/fmicb.2026.1868794

**Published:** 2026-06-30

**Authors:** Suzana Ghosh, Asit Ranjan Ghosh

**Affiliations:** Department of Integrative Biology, School of Bioscience and Technology (SBST), Vellore Institute of Technology (VIT), Vellore, Tamil Nadu, India

**Keywords:** biohydrogenation pathway, CLA, fatty acids, hydratase, LAI, MCRA

## Abstract

Conjugated linoleic acid (CLA) is a group of positional and geometric isomers widely admired for its anti-inflammatory and anti-carcinogenic effects. Enzymes responsible for bioconverting linoleic acid (LA) into CLA are diverse, found in a wide range of microorganisms from lactic acid bacteria (LAB) to non-lactic acid bacteria (non-LAB). Various LAB and non-LAB strains show promising results in CLA production. Enzymes such as linoleate isomerase (LAI), Myosin cross-reactive antigen (MCRA), and oleate hydratase, involved in CLA production, are also diverse in nature. They originate from various sources and have demonstrated potential to biohydrogenate LA and other similar substrates. Comparative analysis of substrate–enzyme dynamics revealed substantial structural conservation alongside functional divergence among various biohyrogenating enzyme-associated pathways, suggesting evolutionary adaptation toward fatty acid detoxification and membrane homeostasis. Furthermore, environmental and physiological factors such as substrate availability, oxygenation, pH, temperature, and growth phase were found to significantly influence CLA biosynthesis. Despite considerable advances, several mechanistic aspects of enzyme cooperation, pathway regulation, and evolutionary relationships remain unresolved. This article critically examines various aspects of substrate–enzyme dynamics, their ecology, evolution, and molecular regulation. It aims to bridge the knowledge gap in catalytic mechanisms and regulation while summarizing available information on various enzymes responsible for the bioconversion of LA to CLA, implicating fundamental microbiology and biotechnology.

## Introduction

Conjugated linoleic acid (CLA) is a group of positional and geometric isomers widely admired for its anti-inflammatory and anti-carcinogenic effects (Mohammadzadeh et al., 2013; Dubey et al., 2016; Dubey et al., 2023). It is also beneficial in managing lifestyle diseases such as obesity (Namazi et al., 2019), type 2 diabetes, and cardiovascular diseases (DeClercq et al., 2012). It therefore has value as an important nutraceutical for human health. However, CLA is an intermediate metabolite in the fatty acid metabolic pathway where linoleic acid (LA) acts as the primary substrate and linoleate isomerase (LAI) is the key enzyme along with its associated enzymes. There are 28 isomers (den Hartigh, 2019), and among them, two familiar isomers of CLA are cis-9, trans-11 and trans-10, cis-12, which have garnered major attention for their potential health benefits (Pariza et al., 2000).

Investigations into LAI have revealed its elaborate mechanisms in governing fatty acid transformations and their implications for various biological processes. LAI and likely other biohydrogenating enzymes take part in bioconversion. These enzymes are produced by several microorganisms where LA and LA-like fatty acids are converted either directly to CLA or via other compounds. On one occasion, CLA is produced via the production of trans-vaccenic acid (TVA) (Salsinha et al., 2018) with a non-microbial enzyme, Δ9-desaturase of the mammary cells (Fukuda et al., 2006). The structural and functional divergence of these biohydrogenating enzymes reflects distinct evolutionary adaptations to diverse ecological and metabolic environments.

The goal of this review is to provide a comprehensive overview of LAI and other biohydrogenating enzymes, delving into their molecular structures, catalytic and regulatory mechanisms governing their expressions within cells, factors influencing activity, and wider implications in biological systems. Moreover, with the integration of insights from biochemical, microbiological, and bioinformatic studies, this review seeks to develop a unified conceptual framework for understanding the molecular architecture of microbial CLA biosynthesis and to highlight future directions for research and biotechnological exploitation. As we explore the various facets of LAI in particular, we will also examine its occurrence and distribution across diverse organisms, shedding light on the evolutionary and ecological dimensions of this enzyme. The biotechnological applications of LAI, particularly in the fabrication of CLA for functional foods and pharmaceuticals, will be discussed, showcasing the relevance of the enzyme(s) in industrial processes.

## Literature search strategy

A comprehensive literature survey was conducted using major scientific databases and search engines such as PubMed, Scopus, and Google Scholar. Relevant articles published mostly between 2000 and 2025 were retrieved using combinations of keywords such as conjugated linoleic acid, CLA biosynthesis, linoleate isomerase, oleate hydratase, MCRA, fatty acid hydratase, biohydrogenation, probiotic, and probiotic enzymes. Additional studies were identified through cross-referencing of relevant articles.

Studies focusing on microbial CLA biosynthesis, enzymatic mechanisms, molecular regulation, evolutionary relationships, structural biology, and biotechnological applications were prioritized and included. Articles lacking sufficient experimental evidence or direct relevance to CLA-producing enzymes were excluded. The collected literature was critically evaluated to provide an integrative overview of substrate–enzyme dynamics, ecological distribution, and evolutionary aspects of CLA biosynthesis.

### Importance of conjugated linoleic acid (CLA)

Trans fats are unsaturated fatty acids with at least one double bond in trans configuration. These are formed in industry by the partial hydrogenation of vegetable oils. Although most known trans fats are associated with various health hazards, CLA are naturally occurring trans fatty acid isomers derived from LA that exhibit several reported health-promoting properties. LAI and associated biohydrogenating enzymes play key roles in the microbial biosynthesis of CLA, contributing significantly in nutritional and health-related contexts. CLA is the name given to a group of geometric and positional isomers of octadecadienoic acid that are generated from LA and contain conjugated double bonds at various positions such as 9, 10, 11, and 12. However, the cis-9, trans-11 and trans-10, cis-12 isomers of CLA are the main biologically active isomers.

CLA has attracted significant attention due to its reported efficiency against cancer. Dubey et al. (2016) showed that CLA produced by *P. pentosaceus* GS4 can successfully and effectively mitigate colon cancer in a murine model (Dubey et al., 2016; Dubey et al., 2023; George and Ghosh, 2025). Clinical studies show that CLA supplementation improves angiogenesis and metastasis by modulating inflammatory markers and reducing matrix metalloprotease (MMP)-2 and MMP-9 (Mohammadzadeh et al., 2013). It regulates the production of cytokines like IL-1β, IL-6, and TNF-*α*, also increasing activation of PPARγ, which in turn modulates immune response leading to the attenuation of inflammation (Changhua et al., 2005).

CLA helps in managing body weight by regulating various parameters such as increasing lipolysis, improving the intestinal probiotic microenvironment, and increasing the synthesis of peroxisome proliferator-activated receptors (PPARs) (Dubey et al., 2023), upregulating the expression of uncoupling protein of respiratory chain-1 (UCP-1). It also aids in managing Irritable Bowel Disease (IBD) (Evans et al., 2010).

CLA has gained increasing evidence in obesity management. Clinical studies suggested that long-term supplementation of CLA by obese middle-aged men with metabolic syndrome may experience a reduction in abdominal fat. However, some studies also demonstrated that the use of trans-10, cis-12 CLA isomer may contribute to insulin resistance in many male individuals. Apart from adults, CLA can lower body fat in obese children (Risérus et al., 2001; Risérus et al., 2002; den Hartigh, 2019). Another study says that long term supplementation of CLA-free fatty acid (CLA-FFA) or CLA-triglycerol helps in reduction of body fat in healthy obese adults (Gaullier et al., 2004).

CLA also inhibits atherosclerosis by lowering blood cholesterol, boosts immunity, enhances liver function, promotes hypoglycemia and facilitates bone density (den Hartigh, 2019; George and Ghosh, 2025). Due to health beneficial claims, CLA containing supplements have gained commercial interest and there is a sustainable market demand.

Despite the reported beneficial effects of CLA on health, several studies show contradictory metabolic and physiological effects. Therefore, CLA should be used in moderate concentrations to prevent harmful effects such as insulin resistance (Ibrahim and El-Sayed, 2021). High-dose intake may lead to increased lipid peroxidation or minor side effects.

### Substrates

Biologically, CLAs are produced through cellular and microbial metabolism involving one or more enzymes, with the presence of several substrates following biohydrogenation pathways. These substrates are essential and/or non-essential fatty acids like linoleic acid (LA), linolenic acid (LNA), oleic acid (OA), and ricinoleic acid (RA).

Linoleic acid (LA) is an essential, omega-6 polyunsaturated fatty acid widely available in many vegetable oils (such as 70–75% in safflower oil, 70% in grapeseed oil, 68–71% in sunflower oil, 51–56% in soybean oil, 15–20% in mustard oil, and 2% in coconut oil). LA primarily functions as the precursor for CLA biosynthesis and hence serves as a substrate for the key enzyme LAI and other associated enzymes. *Propionibacterium* spp. can biohydrogenate LA to trans-10 cis-12 CLA with the involvement of a biohydrogenating enzyme broadly known as PAI (*Propionibacterium acnes* polyunsaturated fatty acid isomerase) (Liavonchanka et al., 2006). LA can either be in free form or esterified with triacylglycerol.

Oleic acid (OA), a monounsaturated (*ω*-9) fatty acid (MUFA) (C18H34O2), is widely found in olive oil (55–80%), canola oil, and sunflower oil, and is treated as a substrate for CLA and CLA-like fatty acid production. Both LA and OA have antimicrobial potential, which may be limited by various surface-active substances, such as lecithin and Tween 80 (Boyaval et al., 1995; Yoon et al., 2018). LA in higher concentration can act as a blocking agent for CLA biohydrogenation. However, supplementation with glycolytic inhibitors like iodoacetate results in a substantial increase in CLA levels (Mei et al., 2021).

Ricinoleic acid (RA) (C_19_H_25_O_3_) (12-hydroxy c9-18:1) is another substrate for CLA biosynthesis. It is predominantly present in castor oil (approximately 88%). Some LAB strains, particularly *Lactobacillus plantarum*, can convert RA to functional CLA (Kishino et al., 2009).

Another fatty acid, trans-vaccenic acid (TVA), is also a precursor of CLA in the LA to CLA conversion pathway. TVA also acts as a substrate for Δ9-desaturase enzymes present in mammalian tissues (Wang et al., 2023).

In research, many vegetable oils were used as a cheaper alternative to LA and RA for bioconversion. Castor oil was employed by Ogawa et al. (2005) as a substrate to produce CLA using LAB. Castor oil is a rich source of triacylglycerol ricinoleic acid. Dubey et al. (2012) showed that sesame oil can be used as a sustainable, alternative substrate source for LA to produce CLA (Dubey et al., 2012), where LA concentration is reported to be between 40 and 50%. Vegetable oils rich in LA are increasingly explored as cost-effective substrates for microbial CLA production. Their fatty acid composition significantly influences conversion efficiency and metabolic yield.

Besides the role of fatty acids, either in pure form or in different oils, as a carbon source for bacterial growth, the carbon source used showed a major change in terms of cell mass and CLA concentration. A study conducted by Razmjooei et al. (2020) showed that CLA concentration was good enough when a 1:1 ratio of lactose and glucose was used in the growth medium (Razmjooei et al., 2020). In another study, it has been reported that when LA is added to the growth medium along with a glycolytic inhibitor like 2-deoxy-D-glucose or 3-bromopyruvate, the outcome shows an appreciable increase in CLA production (Hernandez-Mendoza et al., 2009).

### Biohydrogenating enzymes for CLA production and critical observations

Biohydrogenating enzymes and substrates are significant factors in the production of CLA and CLA-like compounds. Like substrates, several enzymes are involved in the biosynthesis of CLA from diverse microbial sources. Enzymes like linoleic acid isomerase (LAI), oleate hydratase (OleH), and myosin cross-reactive antigen (MCRA) are important.

### Linoleic acid isomerase (LAI)

Linoleate isomerase (LAI) is a crucial microbial biocatalyst that catalyzes the conversion of LA into isomers of CLA. It plays a pivotal role in lipid metabolism, especially in probiotic and gut-associated bacteria. LAI contributes to the bioconversion of LA to biologically active isomers of CLA, which are well known for their health-promoting properties. Due to its industrial and nutritional significance, LAI has gained considerable attention in functional food and biotechnological research.

#### Occurrence and distribution

LAI, an important cellular and microbial enzyme in fatty acid metabolism, exhibits a widespread presence across diverse organisms, showcasing its significance in the complex web of biological processes (Kepler and Tove, 1967). LAI is notably prevalent in bacteria, fungi, and other microorganisms, including human gut microbiota, underscoring its evolutionary significance and functional diversity *(*Póti et al., 2015; Rezavand Hesari et al., 2024). Physicochemical conditions strongly influence the quantity of CLA produced. Few LAB strains produce elevated CLA isomer in the stationary phase (Hernandez-Mendoza et al., 2009). Dubey et al. (2012) showed that *Pediococcus pentosaceus* GS4, a probiotic strain, has the ability to biohydrogenate LA, producing CLA (195 μg/mL) as an intermediate metabolite when supplemented with LA in the stationary phase. Certain *Lactobacillus plantarum* strains act as biocatalysts for CLA production in LA-supplemented media. They can produce up to 40 mg/L of CLA under optimum reaction conditions. It is thus also considered to be the most efficient CLA producer among *Lactobacillus* spp. (Ogawa et al., 2005). In another study, *Lb. casei* has also been reported as a good producer of CLA, producing 838.67 μg/g of CLA from fermented soy milk under optimized conditions (Wang et al., 2022). *Lb. fermentum* DDHI27 also produces CLA in modified skim milk medium. Besides *Lactobacillus* spp., *Bifidobacterium breve* converts LA to CLA (Macouzet et al., 2010). *Bif. breve* and *Bif. dentium* can efficiently produce CLA even in LA non-supplemented medium (Coakley et al., 2003). Some other CLA-producing strains are *Lb. acidophilus*, *Propionibacterium thoenii*, and *Butyrivibrio fibrisolvens* A38 (Kim, 2003). All these studies indicate that CLA biohydrogenation ability is extensively distributed among various probiotic species with varied production efficiency. The variability in CLA production among bacterial strains suggests variation in substrate specificity, enzyme localization, and regulatory architecture. Enhanced production and accumulation of CLA during stationary phase suggests that biohydrogenation may function as a stress-responsive metabolic strategy rather than a constitutive biosynthetic pathway. Moreover, accumulation of CLA during the late growth phase may also contribute to membrane remodeling, oxidative stress tolerance, or detoxification of excess unsaturated fatty acids.

On the other hand, some non-LAB candidates from the genera *Propionibacterium* and *Clostridium* have the potential to produce CLA. *Propionibacterium freudenreichii* subsp. *freudenreichii*, *P. freudenreichii* subsp. *shermanii*, *P. acidipropionici*, *P. tecbnicum* (Jiang et al., 1998) and *P. shermanii* strain JS (Rainio et al., 2002) could produce the cis-9, trans-11 isomer of CLA. *Clostridium bifermentan*s, *C. sporogenes*, and *C. sordelli* produce trans-vaccenic acid (TVA) from LA and CLA as an intermediate (Table 1). Another study reports the CLA-producing property of *Bacillus coagulans* IBRC-M10807 (Rezavand Hesari et al., 2024). Some of these non-LAB strains produce higher amounts of CLA in their early phases of growth.

**Table 1:** List of biohydrogenating enzymes from various microorganisms.

Sl. no.	Name of organism	Protein annotation	Substrate	Isomer(s) produced	References
1	*Propionibacterium acnes (ATCC 6919)*	*P. acnes* linoleic acid isomerase	LA	t 10, c12 CLA	Macouzet et al. (2010)
2	*Lactobacillus acidophilus (ATCC 832)*	LAI (reannotated as MCRA)	LA	t 10,c 12 CLA; c 9, t 11 CLA; trans, trans CLA	Peng et al. (2007)
3	*Bifidobacterium breve LMG 13208 ATCC 15700*	LAI (re-annotated as MCRA)	LA	t10,c12 CLA and trans, trans CLA	Peng et al. (2007)
4	*Lactobacillus curvatus LMG 13553*	LAI	α-LNA	c9, t11, c15; t9, t11, c15 CLNA	Gorissen et al. (2011)
5	*Lactobacillus plantarum ATCC 8014*	LAI	α-LNA	c9, t11, c15; t9, t11, c15 CLNA	Gorissen et al. (2011)
6	*Lactobacillus plantarum IMDO 130201*	LAI	α-LNA	c9, t11, c15; t9, t11, c15 CLNA	Gorissen et al. (2011)
7	*Lactobacillus plantarum LMG 6907*	LAI	α-LNA	c9, t11, c15; t9, t11, c15 CLNA	Gorissen et al. (2011)
8	*Lactobacillus plantarum LMG 13556*	LAI	LA α-LNA	CLA CLNA	Gorissen et al. (2011)
9	*Lactobacillus plantarum LMG 17682*	LAI	α-LNA	CLNA	Gorissen et al. (2011)
10	*Lactobacillus sakei LMG 13558*	LAI	α-LNA	C9, t11, c15; t9, t11, c15 CLNA	Gorissen et al. (2011)
11	*Lactobacillus sakei CGI*	LAI	α-LNA	C9, t11, c15; t9, t11, c15 CLNA	Gorissen et al. (2011)
12	*Bifidobacterium breve* NCIMB 702258	MCRA	LA	10-HOE	Rosberg-Cody et al. (2011a, 2011b)
13	*Streptococcus pyogenes* M49	MCRA	LA	10-Hydroxy and 10,13-dihydroxy derivatives	Volkov et al. (2010)
14	*Bifidobacterium animalis* subsp. *lactis Bb-12*	MCRA	LA and OA	10-HOE and 10-HOA	Yang et al. (2013)
15	*Lactobacillus rhamnosus LGG*	MCRA (reannotated an oleate hydratase)	LA and OA	10-HOE and 10-HOA	Yang et al. (2013)
16	*Lactobacillus plantarum ST-III*	MCRA	LA and OA	10-HOE and 10-HOA	Yang et al. (2013)
17	*Lactobacillus acidophilus NCFM*	MCRA	LA and OA	10-HOE, 10-HOA	Yang et al. (2013)
18	*Lactobacillus delbrueckii* subsp. *bulgaricus* ATCC 11842	OleH	LA	RA	Kuhl et al. (2021)
19	*Lactobacillus reuteri* LTH2584	Linoleate 10- hydratase	LA	10-HOE	Chen et al. (2016)
20	*Lactobacillus acidophilus* NTV001	FA-HY1 FA-HY2	LA	HOE 10-HOE	Hirata et al. (2015)
21	*Lactobacillus plantarum*	α-enolase	LA	c 9, t 11 CLA	Ortega-Anaya and Hernández-Santoyo (2016)
22	*Lactobacillus spicheri* LP38	Linoleate 10- hydratase	LA	10-HOE	Chen et al. (2016)
23	*Lactobacillus hammesii* DSM 16381	Linoleate 10- hydratase	LA	10-HOE	Chen et al. (2016)
24	*Lactobacillus plantarum* TMW1.460	Linoleate 10- hydratase	LA	10-HOE	Chen et al. (2016)

Beyond bacterial systems, several strains of yeast also have the ability to biohydrogenate LA to CLA. *Saccharomyces cerevisiae* first showcased the ability to produce CLA among yeast (Hornung et al., 2005). *Yarrowia lipolytica*, an oleaginous yeast, also has the capacity to produce CLA due to the presence of LAI (Imatoukene et al., 2017).

Some species of algae likely produce raw material essential for CLA biosynthesis, such as *Schizochytrium* spp. Specific red algae, like *Ptilota filicina*, have the potential to produce CLA. *P. filicina* possesses a novel enzyme, polyenoic fatty acid isomerase, which is able to produce conjugated trienes from different polyunsaturated fatty acids (Wise et al., 1994).

In mammals, the mammary glands of ruminants, mice, and humans (Santora et al., 2000; Turpeinen et al., 2002; Wang et al., 2022) can produce CLA from TVA using Δ9 desaturase (SCD1). Another desaturating enzyme, Δ13 desaturase, present in the mammary tissue of both ruminants and rodents, can introduce a double bond at the C-11 and C-13 positions of LA. This produces the trans-11, cis-13 CLA isomer and is encoded by the fatty acid desaturase 3 gene (Garcia et al., 2017). Thus, the enzymes responsible for CLA production are diverse and phylogenetically widespread. This emphasizes their ecological importance for fatty acid detoxification and membrane homeostasis under diverse environmental conditions, depicting the impact of these enzymes on both microbial communities and higher organisms.

#### Structure and function

Studies indicate that LAI may exist either as a soluble cytoplasmic enzyme, as reported in *P. acnes*, or as a membrane-associated protein in several other microorganisms (Farmani et al., 2010). An insoluble form of the enzyme, possibly due to its hydrophobicity, has also been reported elsewhere (Kepler and Tove, 1967).

**Figure 1:**
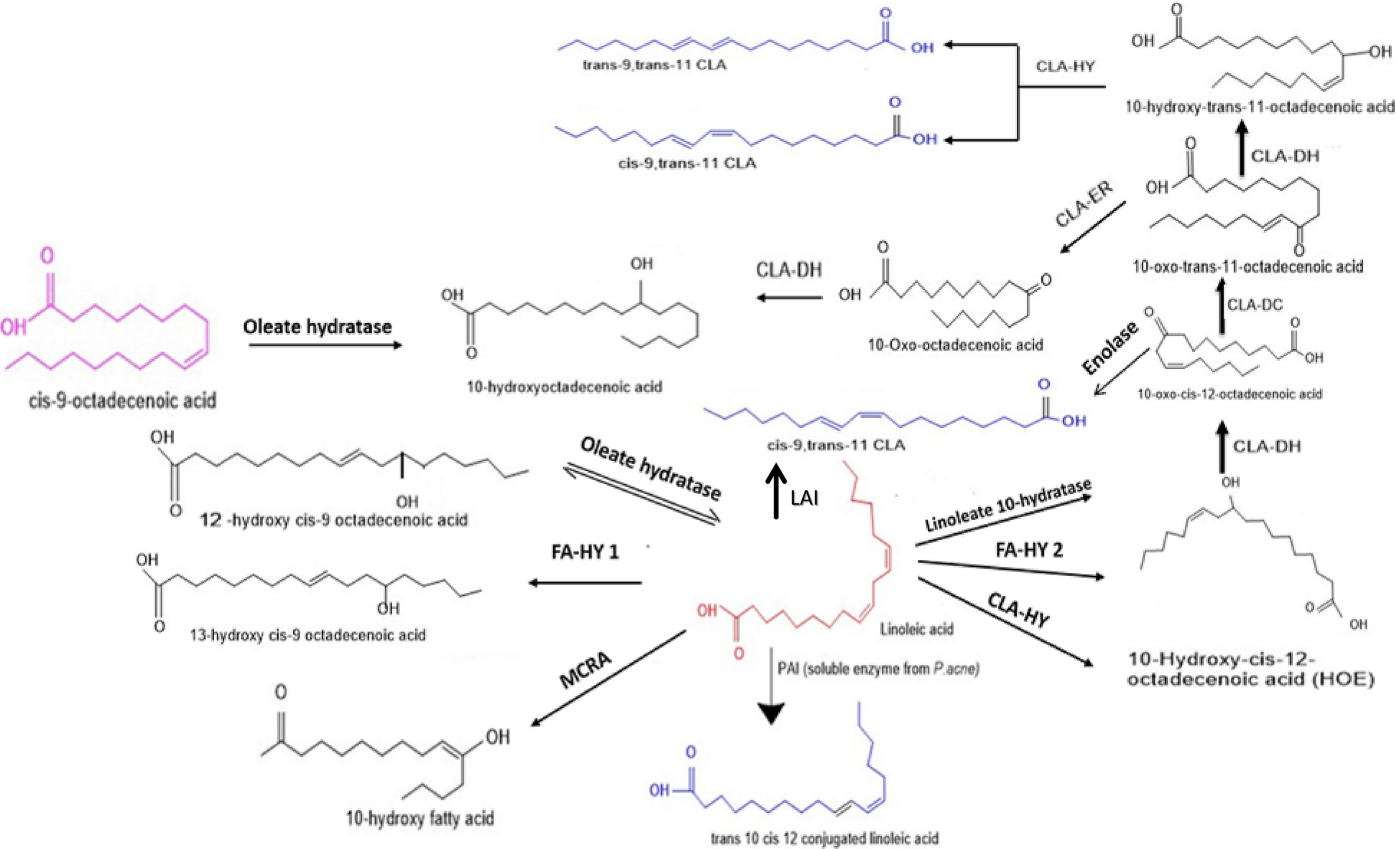
Schematic diagram showcasing the microbial conversion of CLA. Various microbial enzymes are responsible for the bioconversion of CLA and hydroxy fatty acids from polyunsaturated fatty acids (PUFAs), such as linoleic acid (LA) and oleic acid (OA). CLA-HY, CLA oleate hydratase; CLA-DH, CLA short-chain dehydrogenase; CLA-DC, CLA acetoacetate decarboxylase; CLA-ER, CLA-enone reductase; MCRA, myosin cross-reactive antigen, FA-HY 1/2, fatty acid hydratase 1/2.

To study membrane-bound characteristics of LAI, scientists studied selected genera, *Lactobacillus*, *Bifidobacterium*, and *Leuconostoc* by genome and amino acid sequencing. Analyses revealed its membrane-bound characteristics with the existence of certain conserved patterns, including a presumed flavin adenine dinucleotide (FAD) binding region (Macouzet et al., 2010). The identification of conserved membrane-associated motifs and putative FAD-binding regions suggests that membrane localization may facilitate substrate accessibility and enhance catalytic efficiency during fatty acid biohydrogenation. Kishino et al. (2011a) confirmed a novel multi-component aspect of LAI in *Lb. plantarum* species. They showed two fractions, membrane-bound and soluble, that are responsible for the synthesis of conjugated fatty acids. There are two steps for CLA formation: i) hydration of LA to 10-hydroxy-12-octadecenoic acids (10-HOE) and ii) dehydrating isomerization of 10-hydroxy-12-octadecenoic acids to CLA. Hydration and dehydration are performed by the membrane fraction. Combined efforts of the soluble and membrane parts cause the conversion of CLA from either LA or 10-HOE (Kishino et al., 2011b). Liu et al. (2021) also provided information in support of a multi-component enzyme system in *Lb. plantarum*. They demonstrated that the activation of the operon system was responsible for CLA production as an intermediate in the presence of LA-supplemented medium. Operon-dependent regulation further suggests that CLA biosynthesis is tightly coordinated at the transcriptional level in response to substrate availability and environmental conditions. LAI uses FAD as a cofactor (Kishino et al., 2011a, 2011b), which further confirms that NADPH/NADH along with FAD restores complete enzymatic activity. In another study, Kishino et al. (2013) showed that LA was transformed into cis-9, trans-11 CLA (CLA1), trans-9, trans-11 CLA (CLA2), trans-10 octadecenoic acid, and oleic acid (OA) using the corresponding catalysts: CLA-HY (CLA oleate hydratase), CLA-DH (CLA short-chain dehydrogenase), CLA-DC (CLA-acetoacetate decarboxylase), and CLA-ER (CLA-enone reductase) (Figure 1). These findings illustrate that LA metabolism in *Lb. plantarum* involves several sequential reactions. Initially, LA undergoes hydration of its carbon–carbon double bond (C=C) at the Δ9 position catalyzed by CLA-HY, resulting in the formation of a 10-hydroxy fatty acid (Figure 1). Subsequently, the hydroxy group (OH) at C10 is dehydrogenated by CLA-DH, yielding a 10-oxo fatty acid. Following this, the C=C at Δ12 is isomerized by CLA-DC, leading to the creation of a conjugated enone structure, namely, 10-oxo-trans-11 fatty acid. Next, CLA-ER catalyzes the hydrogenation of the C=C at Δ11, converting it into a carbon–carbon single bond (C-C). Then, CLA-DH facilitates the hydrogenation of the oxo group at C10, producing another 10-hydroxy fatty acid. Lastly, CLA-HY catalyzes the dehydration of OH at C10, resulting in the generation of cis-9 and trans-10 monoenoic fatty acids. These structural elements act in concert to facilitate the interaction of the enzyme with LA and orchestrate its subsequent isomerization into CLA (Kishino et al., 2013). Altogether, these findings indicate that CLA biosynthesis in *Lb. plantarum* involves a coordinated multi-enzyme system comprising hydration, oxidation, isomerization, reduction, and dehydration reactions. The sequential cooperation of membrane-associated and soluble enzyme fractions suggests a highly regulated metabolic network rather than the activity of a single catalytic protein.

Another study by Ortega-Anaya and Hernández-Santoyo (2016) demonstrated the function of *α*-enolase in the biohydrogenation pathway. They are of the opinion that this α-enolase belongs to the enolase family generally related to glycolysis. It cannot directly utilize LA; rather, it uses the hydroxyl derivative of LA, 10-HOE, to produce bioactive cis-9, trans-11 CLA in *Lb. plantarum* (Ortega-Anaya and Hernández-Santoyo, 2016).

The catalytic activity of LAI hinges on the molecular intricacies of its active sites and the coordinated interaction of key amino acid residues involved in substrate binding and isomerization. These sites serve as molecular orchestrators, guiding the complex series of biochemical reactions that lead to the isomerization of LA. Within these active sites, amino acid residues play a crucial role in mediating the molecular rearrangements necessary for the transformation of LA into CLA. A thorough exploration of these catalytic mechanisms is essential for unraveling the specific role of the enzyme in the complex functionality of fatty acid metabolism.

Beyond the catalytic core, the molecular structure of LAI is often characterized by distinctive domains or pockets. These structural features play a critical role in binding LA with specificity, underscoring the selectivity of the enzyme toward its substrate. Unraveling the molecular basis of this selectivity provides crucial insights into the substrate interaction of LAI and enhances our understanding of its role in shaping cellular fatty acid profiles.

A study conducted by Volkov et al. (2013) used the model structure of *Lb. acidophilus* (PDB ID 4IA6) as a template. LA was observed bound within a cavity that functions as an entrance portal for fatty acids (Volkov et al., 2013). A tunnel assay indicated a potential connection between the cavities responsible for binding fatty acid and FAD, suggesting a pathway for fatty acid transport toward the FAD-binding site. The FAD-binding site was identified by the presence of a conserved signature motif GXGXXG(X)17/23E (Volkov et al., 2013).

Despite substantial progress in identifying catalytic motifs and structural domains, the precise mechanism, membrane interaction, and substrate specificity remain poorly understood. The coexistence of soluble and membrane-associated LAI systems across different microorganisms suggests possible evolutionary divergence in catalytic adaptation and lipid utilization strategies.

### Oleate hydratase (OleH)

The genome sequencing data of *Lb. delbrueckii* subsp. *bulgaricus* LBP UFSC 2230 unveiled a solitary gene responsible for encoding an enzyme, oleate hydratase (*OleH*), in contrast to the usual presence of multiple genes in analogous strains (Kuhl et al., 2021). This research delved into the biological impact of the OleH enzyme from *Lb. delbrueckii subsp. bulgaricus* LBP UFSC 2230 on LA hydration and ricinoleic acid (RA) dehydration, and its potential involvement in CLA production. Through three-dimensional homology modeling analysis, it was observed that the *Lb. delbrueckii subsp. bulgaricus* LBP UFSC 2230 OleH possesses two potential fatty acid-binding sites. Docking analysis of this OleH indicated nonpolar interactions involving residues G31, A33, and E57, suggesting their involvement in the FAD binding domain within the conserved motif G^29^G^30^G^31^L^32^A^33^G^34^(X)_23_E^57^ (Kuhl et al., 2021). The presence of a single OleH-encoding gene may indicate a more specialized or tightly regulated fatty acid biohydrogenation system in this strain.

Interestingly, a reversible transition from LA to RA, and subsequently to LA and finally to CLA, has been observed in some seminal studies. Gao et al. (2019) found a reversible reaction in the MCRA of *Bifidobacteria*, otherwise called fatty acid hydratase, that has the ability to convert 9-hydroxy-cis-12-octadecenoic acid (ricinoleic acid, RA) into LA in a reversible manner. Subsequently, LA is further transformed into CLA through the action of LAI. The Oleate hydratase (OleH) from *Lb. delbrueckii* subsp. *bulgaricus* LBP UFSC 2230 also has the capacity to perform reversible transition of LA to RA, as the enzyme is capable of both hydration of LA and dehydration of RA (Kuhl et al., 2021).

Oleate hydratase (OhyA, or OleH, as mentioned in some other studies) is also a flavoenzyme bearing an FAD motif which is critical for the function of the enzyme (Hiseni et al., 2015). Therefore, FAD-mediated redox interactions are central to substrate recognition and catalytic efficiency in OleH-associated biohydrogenation. This dependency on FAD also indicates that electron transfer mechanisms are intimately related to hydration-mediated fatty acid modification during early stages of CLA biosynthesis. The OhyA catalyzes hydration of an unsaturated fatty acid to its corresponding hydroxyl-fatty acid (Radka et al., 2021). OhyA functions as a forerunner of CLA formation by catalyzing it in its initial stages (Yang et al., 2013). OhyA belongs to the MCRA family (Rosberg-Cody et al., 2011a) as it has a Rossman fold and belongs to the NADP Rossman protein clan (Pfam clan entry CL0063) suggesting its nucleotide binding capacity (Bhattacharyya et al., 2012). Furthermore, Oleate hydratase is found in 50 metabolic pathways suggesting its importance in diversity and relevance in the field of microbial lipid metabolism (Zhu et al., 2024). Despite advances in structural and docking analyses, the precise correlation between OleH conformational dynamics, membrane localization, and substrate specificity remains unclear. Further biochemical and crystallographic studies are required to clarify how structural flexibility influences reversible fatty acid transformation during CLA biosynthesis.

### Myosin cross-reactive antigen protein (MCRA)

MCRA proteins, as the name suggests, have the potential to bind to myosin antigens. They were first encountered in *Streptococcus pyogenes* M49 with PUFA isomerase activity. The 67-kDa MCRA of *S. pyogenes* M49 was isolated and sequenced. Sequence analysis revealed similarity and homology of the MCRA protein to the beta chain of MHC II in humans and mice, according to protein database comparison, suggesting possible structural conservation beyond lipid metabolic functions. Structural analysis identified the presence of an N-terminal hydrophilic domain in the MCRA protein, followed by a middle hydrophobic domain and a polar C-terminal end (Kil et al., 1994). Subsequent studies established the role of MCRA as a fatty acid hydratase involved in the conversion of LA into 10-HOE and 10, 13-dihydroxyoctadecenoic acid (Volkov et al., 2010).

The prevalence of MCRA protein enabled various microbes to overcome several stressful conditions when exposed to different compounds such as ethanol, butanol, acetate, and salts like sodium lactate and sodium chloride. In *Lb. acidophilus*, the putative MCRA protein encoded by the LBA649 gene increases microbial tolerance, allowing the cell to withstand such stressful conditions (Salsinha et al., 2018). This provides an adaptive mechanism for membrane stabilization and detoxification of unsaturated fatty acids, contributing to broader cellular strategies. Ogawa et al. (2001 and, 2005) reported the initial documentation of 10-HOE accumulation during CLA production by *Lb. acidophilus* and also proposed a pathway involved in hydration and dehydration steps. *Lb. acidophilus* AKU 1137 produced 10-hydroxy-cis-12-octadecenoic acid (10-HOE) when incubated in LA-supplemented MRS broth (de Mann Rogosa Sharpe) for the first two days, which slowly and eventually converted to CLA.

Kishino et al. (2011a, 2011b) documented the presence of multiple-component enzymes involved in the production of CLA from *Lb. plantarum* AKU1009a, as mentioned before. Through ultracentrifugation and separation of fractions, a membrane-bound protein was identified as responsible for 10-HOE production. Additionally, when the membrane fraction was combined with two unidentified proteins from soluble fractions, the synthesis of cis-9, trans-11 CLA was observed. CLA was produced as a result of a multi-step reaction where LA is first converted to 10-HOE, which then undergoes dehydration and isomerization of the double bond to 10-oxo-trans-11-octadecenoic acid, followed by rehydration and final conversion to CLA (Kishino et al., 2011a, 2011b).

Yang et al. (2017) studied lactic acid bacteria such as *Lb. acidophilus*, *Lb. brevis*, *Lb. bulgaricus*, *Lb. casei*, *Lb. plantarum*, *Lb. rhamnosus*, *Lb. reuteri*, and *Bif. animalis*. They showed that MCRA was amplified and helped in the hydration of LA to 10-HOE, indicating its role as a fatty acid hydratase (Yang et al., 2017).

**Figure 2:**
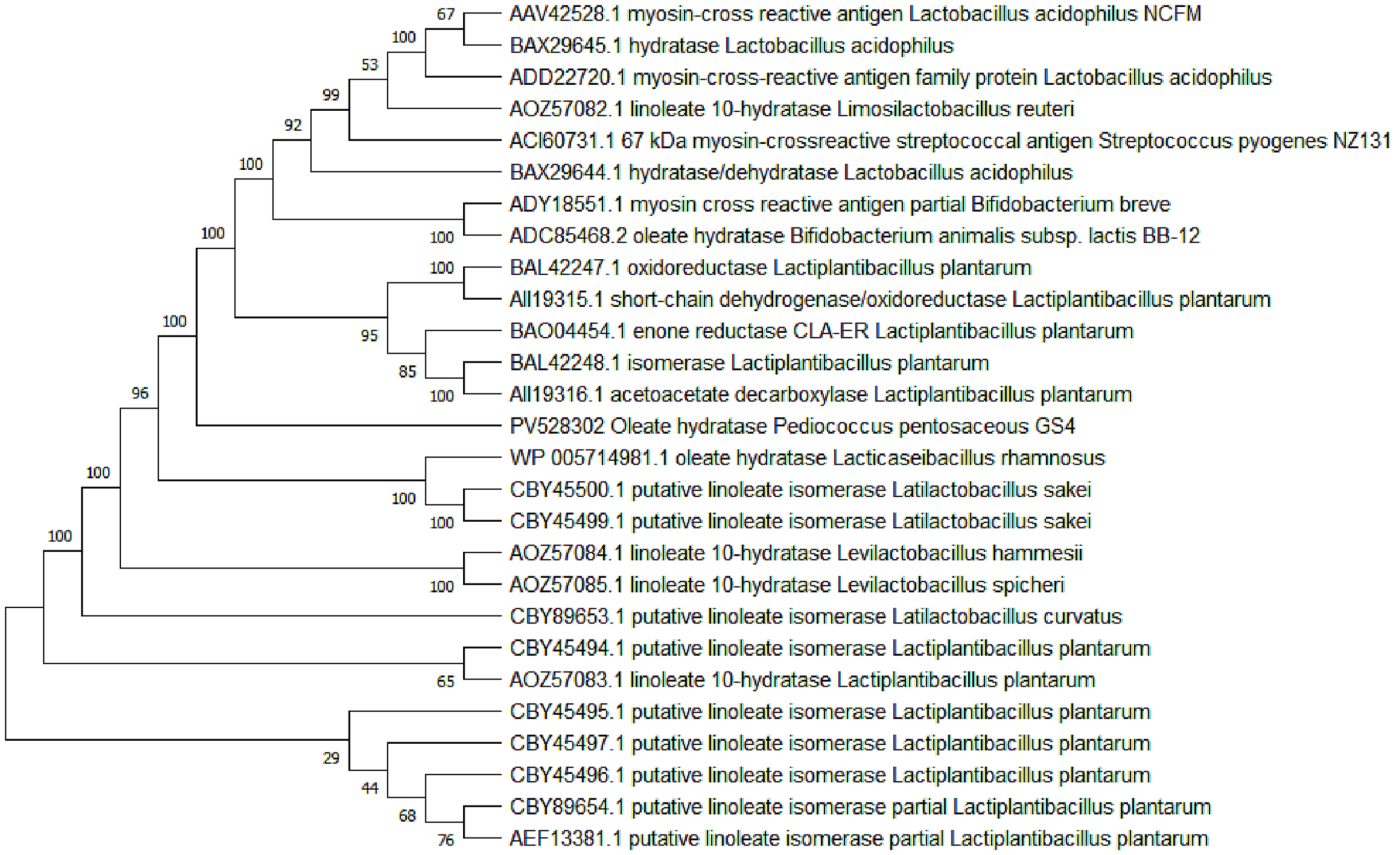
An unrooted phylogenetic tree constructed based on CLA hydrogenating enzymes from various bacterial species. Sequences were obtained from NCBI with specified organism names. Numbers at the nodes indicate the levels of bootstrap support based on neighbor-joining analysis of 1,000 resampled data sets. Bootstrap values below 50% are not shown. The scale bar represents 0.2 substitutions per nucleotide position. The protein sequences of all reported isomerase, MCRA, and hydratase enzymes were retrieved from the GenBank database, and a multiple sequence alignment was performed using ClustalW. The phylogenetic tree was constructed using the neighbor-joining algorithm by MEGA 11.

Two independent studies by Rosberg-Cody et al. (2007) and Volkov et al. (2013) confirmed the presence of FAD-containing hydratase in MCRA protein. All these studies thus conclude that MCRA family proteins function as FAD-dependent fatty acid hydratase involved in the synthesis of CLA precursors. Volkov et al. (2013) also observed heterogeneous expression of MCRA proteins of *S. pyogenes* in *E.coli*, which worked as a hydratase but showed no CLA-forming isomerase activity, suggesting MCRA alone may not be sufficient for complete CLA biosynthesis. This observation supports the hypothesis that CLA production requires the coordinated action of multiple enzymes rather than a single catalytic protein. An interesting study conducted by Roseberg-Cody and group in 2011 involved cloning the MCRA protein found in *Bif. breve* NCIMB 702258 in *Lactobacillus* spp. and *Corynebacterium* spp. They concluded that *Bif. breve*-derived MCRA behaves like an FAD-containing hydratase enzyme, which catalyzes the preliminary step in CLA production. The authors observed that bacteria expressing this gene have excess 10-HOE and 10-hydroxy-octadecanoic acid (10-HOA) in the spent medium. Some studies indicate that MCRA is a type of oleate hydratase involved in stress tolerance (Rosberg-Cody et al., 2011b). From an evolutionary point of view, MCRA is found to be more closely related to the hydratase group than LAI enzymes, thus concluding hydration reaction as the ancestral step in CLA biohydrogenation pathways (Figure 2).

### Fatty acid hydratase (FA-HY)

The function of hydratase enzymes is the hydration and dehydration of carbon–carbon double bonds in unsaturated fatty acids (Demming et al., 2018). FA hydratase activity has been observed in several MCRA proteins, primarily in relation to *Lactobacillus* spp. It was demonstrated in a study that the gut bacteria *Lb. acidophilus* NTV001 have a high capacity to synthesize 13-hydroxy-cis-9-octadecenoic acid (13-HOE) from LA (Hirata et al., 2015). Fatty acid hydratase 1 (FA-HY1) and FA-HY2 were discovered during those investigations. FA-HY2 stimulates the synthesis of 10-HOE, and FA-HY1 is responsible for producing 13-HOE from LA. FA-HY1 and 2 were identified as members of the MCRA protein family, having FAD binding motifs located in the N-terminal region of the proteins. Thus, it shows a tight evolutionary relationship between MCRA and hydratase proteins (Hirata et al., 2015).

Oleate hydratases (OleH, ohyA) and linoleate isomerases (LAI) are enzymes that operate on similar substrates and exhibit significant sequence similarity. Despite their close resemblance, they perform distinct biochemical reactions. Similarly, LAI was found to have 99.5% identity with *Lb. acidophilus* hydratase (LAH) (Fibinger et al., 2016), but they showed divergence in function. LAH facilitates the addition of water to substrates, while LAI is responsible for the isomerization of double bonds. Due to a difference in only three amino acid residues, the functional disparity between LAH and LAI underscores the subtle yet crucial differences in their catalytic activities.

*Lb. plantarum* has been shown to express a linoleate 10-hydratase enzyme (10-LAH), leading to the production of antifungal 10-HOE with concurrent 13-HOE activity. Comparative analysis of this gene with four other genes encoding putative LAHs from Lactobacilli (*Lb. reuteri* LTH2584, *Lb. plantarum* TMW1.460, *Lb. hammesii* DSM16381, and *Lb. spicheri* Lp38), revealed the presence of FAD-dependent 10-LAHs within the MCRA family (Chen et al., 2016). Intriguingly, there was no clear distinction between 10-LAH and linoleate 13-hydratase (13-LAH), or enzymes capable of producing both 10-HOE and 13-HOE, based on phylogenetic and sequence analyses (Chen et al., 2016). This indicates that significant genetic similarity or close phylogenetic relationships are insufficient for accurately predicting enzymatic function. Consequently, studies focusing on recombinant protein expression can provide valuable insights.

Furthermore, understanding the structural and mechanistic relationships between CLA and hydroxy fatty acid formation remains limited (Kishino et al., 2011a, 2011b). However, the crystal structure of a LAH has been characterized, revealing a homodimeric protein structure with each protomer consisting of four domains. Three of these domains form the FAD and substrate binding sites. The fourth domain, located at the C terminus, covers the entrance to the hydrophobic substrate channel. This domain shows flexibility and undergoes conformational changes by displacing the terminal helices pair in the presence of LA, facilitating the entry of the substrate into the hydrophobic binding channel. Such conformational flexibility likely contributes to substrate specificity and catalytic regulation during microbial fatty acid biohydrogenation. The study confirmed that LAH is active only toward free fatty acids and requires a cis-9 double bond. Additionally, among the identified homologous structures, PAI was found to share a similar biological function with LAH (Volkov et al., 2013). Despite advances in structural characterization, the precise molecular mechanism governing the transition between hydratase and isomerase activity remains poorly understood. Deeper research and further biochemical and crystallographic investigation are required to understand the interplay between active-site dynamics, FAD interactions, and substrate orientation.

### Homology and phylogenetic analysis

Further research conducted by Macouzet et al. (2010) revealed that the coding region of the presumptive linoleate isomerase gene of *Bif. breve* LMG 13208 is extremely similar to the gene cluster of *Lb. acidophilus* (ATCC 832), *Lb. gasseri* (ATCC 33323), and *Leuconostoc mesenteroids* (ATCC 8293), but shows approximately 53% homology with the sequence of the presumptive LAI gene of *Lb. reuteri*, which was shown to be the highest producer of cis-9, trans-11 in this study. The relatively low sequence homology between *Lb. reuteri* and other putative LAI genes, despite its high CLA-producing capability, suggests that catalytic efficiency may depend more on active-site architecture and regulatory mechanisms than on overall sequence conservation.

The authors also predicted the protein of the presumptive LAI gene and showed that it is an integral membrane peptide, consisting of 625 amino acid residues with a molecular mass of 70.52 kDa and an isoelectric point of 5.42. To delve into the structural, functional, and phylogenetic relationships of LAIs, as well as to explore the recombinant production of the target enzyme, Farmani et al. (2010) utilized a variety of bioinformatics tools to analyze their primary structure and physicochemical characteristics (Farmani et al., 2010).

The study revealed that LAIs can be categorized into four distinct classes. The enzyme extracted from *P. acnes*, PAI, has been classified as Group 1 (G I) as there is a scanty correlation between PAI and other LAIs. Group II comprises LAI from *Lb. reuteri* and *Lb. acidophilus*. However, the MCRA from *Lb. acidophilus* NCFM has been denoted as (*Lb. acidophilus* hydratase) LAH, highlighting the functional overlap and evolutionary ambiguity between hydratase and LAI enzyme families (Volkov et al., 2013; Yang et al., 2013). The Group III (G III) LAI bacteria are *Lb. lactis subsp. lactis*, and Group IV (G IV) comprises *Bif. dentium* and *Bif. breve* LAIs (Table 2). Each of these LAI proteins also displays variation in product specificity. PAI functions as a C18:2 t10, c12 isomerase, whereas MCRA-like LAIs primarily act as RA isomerases. The authors, with the aid of bioinformatics tools, revealed the potential sites of glycosylation in LAI, suggesting additional layers of structural regulation (Farmani et al., 2010). The increased similarity between MCRA and LAI was reported in *Lb. reuteri* PYR8 (Rosberg-Cody et al., 2011b). Alternatively, LAI enzymes produced in various bacterial strains could be segregated into two separate families, namely, those resembling *P. acnes* and those akin to myosin cross-reactive antigen (MCRA), each exhibiting distinct isomerase activities.

**Table 2:** Proposed groups of linoleic acid isomerases (LAIs) using *in silico* analysis (Farmani et al., 2010).

Group (G)	Enzyme	Product	Nature	Organism
G I	PAI (linoleate isomerase)	18:2, 10 E 12 Z	Soluble	*Propionibacterium acnes*
G II	LAI (Linoleate isomerase)	18:2, 9Z 11E 18:2, 9Z 11E. 18:2, 10 E 12Z	Membrane bound	*Lactobacillus reuteri* *L. acidophilus*
G III	MCRA(LAH)	18:2, 9Z 11E 18:2, 9Z 12Z	Membrane bound	*L. plantarum*, *Rhodococcus erythropolis*
G IV	MCRA	18:2, 9Z 12Z 18:2, 9Z 11E	Membrane bound	*Bifidobacterium dentium*, *B. breve*

### Ecological and evolutionary adaptation of CLA-producing enzymes

CLA biohydrogenating enzymes are distributed among microorganisms inhabiting diverse ecological niches, including the gastrointestinal tract, rumen ecosystem, fermented foods, and environmental habitats. In these environments, unsaturated fatty acids such as LA may exert antimicrobial and membrane-disrupting effects. Consequently, the ability to metabolize polyunsaturated fatty acid through enzymes such as LAI, MCRA, and OleH likely provides a selective advantage by reducing fatty acid toxicity and maintaining membrane homeostasis. Therefore, differences in substrate availability and environmental conditions may act as ecological drivers shaping the evolution and diversification of CLA-producing enzymes.

Structurally similar proteins with different functions are produced by gene duplication, rearrangement, insertion, deletion, or substitution of amino acids and subsequent sequence divergence (Bartlett et al., 2003). Conversely, some proteins that are structurally homologous but differ in functionality evolve from non-homologous gene sequences, hence differing in sequence and structure (Leppänen et al., 1999). Functional divergence occurs when gene duplication and sequence divergence generate structurally similar and functionally different proteins (Glasner et al., 2006; Chiang et al., 2008). Conversely, some proteins originating from non-homologous gene sets show similar (or the same) function on the same or similar substrate but differ in structures, demonstrating convergence of function. Bacteria undergo such changes to combat various xenobiotic challenges (Russell et al., 2011). A comparable example of functional divergence despite structural conservation is observed within the enolase superfamily: muconate-lactonizing enzyme (EC 5.5.1.1) and mandelate racemase (EC 5.1.2.2) were the first enzymes identified to show considerable structural homology but catalyze different reactions (Cuesta et al., 2014).

Isomerases are ubiquitously present in the genomes of almost all living organisms and hold a crucial role in cellular metabolism. Isomerization comprises 4% of the total biochemical reactions in central metabolism, offering an indispensable role in carbohydrate metabolism along with terpenoid metabolism. It also shows a critical role in the bioconversion of free fatty acids to conjugated fatty acids in the biohydrogenation pathway. The percentage of isomerase present varies among species; it is 2.6% in humans, whereas this ratio increases to 6.2% in bacterial genomes such as *E. coli* (Cuesta et al., 2014).

Isomerases frequently alter the overall chemistry of their substrates while maintaining the structure, rather than preserving the chemistry and changing the substrates. LAI belongs to a fascinating group of enzymes that have diverged functionality while maintaining significant sequence homology. Phylogenetic analyses reveal distinct evolutionary patterns that help us understand the origins and functional diversity of these enzymes.

One of the significant evolutionary relationships between LAI and MCRA proteins is that they share substantial sequence similarity. The LA from *Lb. acidophilus* and *Lb. reuteri* PYR8 exhibits more than 50% homology with the MCRA protein from *Bif. breve*. This prominent similarity points to a shared ancestor protein that underwent divergence to serve distinct roles in several bacterial species. Despite appearing to be more evolutionarily related to the hydratase group, phylogenetic study shows that MCRA proteins branch between LAI and oleate hydratase families (Rosberg-Cody et al., 2011b). Given their location in the evolutionary tree, MCRA proteins might be a distinct branch that descended from a common ancestor or an intermediate evolutionary stage, utilizing the same catalytic cofactor, FAD. This functional divergence despite structural similarities is an iconic illustration of how enzymes can develop distinct catalytic capabilities while preserving similar overall protein architecture. Evolutionary analysis of the oleate hydratase (OhyA, OleH) protein family is an intriguing example of molecular convergence and divergence, where enzymes that catalyze the same biochemical reaction have developed distinct structural and functional characteristics. This highlights that gene regulation is an important criterion for understanding the evolution of the OhyA protein family (Neff et al., 2024). Despite having structural similarities to a number of flavoenzymes, such as tryptophan 5-halogenase, tetracycline 7-halogenase, and monoamine oxidase, oleate hydratase is unique in that it binds unsaturated fatty acids (Hagedoorn et al., 2021). MCRA, OleH, and LAI from various organisms catalyze similar reactions, suggesting convergence of function (Figure 2). Collectively, these findings suggest that microbial fatty acid hydrogenating enzymes have undergone extensive evolutionary diversification driven by substrate availability, membrane adaptation, and ecological stress responses. Such evolutionary flexibility likely contributed to the emergence of structurally related yet functionally specialized CLA-producing systems. The structural conservation yet functional divergence among LAI, MCRA, and OleH proteins reflects evolutionary pressure for substrate adaptation in different ecological niches. Such divergence likely enabled microorganisms to optimize fatty acid metabolism in response to varying environmental lipid compositions and stress conditions. Hence, the available reports suggest that substrate–enzyme interactions are closely linked to both ecological adaptation and molecular evolution. Variations in substrate availability, environmental stress, and membrane requirements may have promoted the diversification of CLA-producing enzymes across microbial taxa. Thus, the structural conservation and functional divergence observed among LAI, MCRA, OleH, and fatty acid hydratase reflect evolutionary responses to distinct ecological niches.

### Co-factors and conserved motifs

Protein sequence-structure analysis of various biohydrogenating enzymes showed the presence of conserved motifs such as FAD-binding region. The presence of putative FAD domain (Macouzet et al., 2010) and a Flavin binding domain at the N terminus (Rosberg-Cody et al., 2011b) has been documented in MCRA protein of *Bif. breve*. The FAD-binding site was identified by the conserved signature motif GXGXXG(X)_17/23_E. Volkov et al. (2013) proposed the possible function of FAD motif is for substrate activation and hydrogen transfer. NADP and NADPH together with FAD enable the protein to be fully functional (Kishino et al., 2011a, 2011b).

As mentioned earlier, LAI shows remarkable sequence homology with OleH (EC 4.2.1.53). Though they show activity on similar substrates, the product formed differs considerably. FAD as an intrinsic co-factor is prevalent in both groups of enzymes, appearing to have distinct roles in each enzyme. In the case of isomerases, a redox-based mechanism has been identified, whereas only a structural or charge stabilizing role has been proposed for hydratases (Fibinger et al., 2016). The formed *trans*-double bond fatty acids or hydroxyl fatty acids are destined to undergo *β*-oxidation.

### Regulation and expression

The precise regulation of LAI is crucial for maintaining cellular lipid homeostasis and orchestrating responses to fluctuating environmental conditions. The expression levels and activity of LAI are tightly controlled by intricate regulatory mechanisms, ensuring a dynamic response to varying metabolic demands and external cues.

Liu et al. (2021) reported the presence of an operon system for CLA production in *Lb*. *plantarum*, which encodes the enzymes CLA-HY, CLA-DH, and CLA-DC. LTTR, a transcription factor from the LySR family, regulates this operon. Attachment of LTTR to the regulator binding motif (TTAAAAGTACTAA) causes the activation and transcription of *cla-dh* and *cla-dc*. Thus, CLA concentration can be adjusted by regulating LTTR. In a different study on *P. acnes* conducted by Liu et al. (2023), it was revealed that increasing the expression of *gdhA* (glutamate dehydrogenase gene) facilitates the transformation of LA into CLA.

It is a glutamate dehydrogenase that specifically utilizes NADP and plays a role in the metabolism of glutamate. Increasing the expression of acetyl CoA is also advantageous for the accumulation of unsaturated fatty acids. Hence, the expression of LAI is often under the control of genetic regulatory elements within the organism’s genome (Liu et al., 2023). Promoter regions and transcription factors play key roles in modulating the synthesis of LAI at the transcriptional level. Understanding the genetic regulation provides insights into how cells fine-tune the expression of this enzyme in response to specific physiological requirements. *Y. lipolytica* was genetically manipulated for elevated CLA production; when produced and accumulated triglycerides were eliminated, overexpression of the *Δ*-12 desaturase enzyme resulted in increased CLA production (Imatoukene et al., 2017).

## Culture conditions

### Oxygenation pattern

*Lb. reuteri* exhibits a preference for micro-aerobic conditions over aerobic ones for growth, although there is enhanced LA bioconversion in oxygen-rich environments. Kim (2003) observed that LA was predominantly converted into hydrogenated products when an anaerobic culture of washed *Bif. fibrisolvens* cell suspension was incubated, and minimal CLA was detected. Both *Lb. reuteri* and *Bif. fibrisolvens* demonstrated increased CLA production and accumulation when they were incubated aerobically (Kim, 2003).

#### pH and temperature optima

Another two extrinsic parameters that can critically affect the isomerization activity of LAI are the temperature and pH of the medium. These two parameters are absolutely crucial for the formation of CLA and CLNA (conjugated linolenic acid, derived from linolenic acid). A study conducted by Gorissen et al. (2011) showed that the highest concentrations of CLA (0.02 mg/mL) and CLNA (0.08 mg/mL) were yielded at 30 °C and at pH 6.2 by the homofermentative *Lb. sakei* LMG13558 after 16 h of culture. At 25 °C and pH 6.2, the amount of CLA accumulation decreased when compared with 30 °C. Maintaining the same pH and decreasing the temperature further to 20 °C, the concentrations of cis-9 trans-11 CLA isomer and trans-9, trans-11 CLA isomer were 0.05 mg/mL and 0.02 mg/mL, respectively. This could be attributed to the lower temperature, which possibly changed the composition of the cellular membrane. *Lb. reuteri* produces a maximum of 0.108 mg/mL of CLA upon incubation of more than 30 h in MRS broth supplemented with LA and the temperature maintained at 10 °C. The authors did not notice any notable elevation in the aggregation of cis-9, trans-11 CLA isomer after 30 h of incubation. At 22 °C, *Lb. reuteri* produced the next highest intensity of CLA. The study concluded that the highest CLA production occurred when LA concentration was low and the temperature varied from 10–20 °C. When the temperature was increased to 22 °C, a 40% depreciation in CLA production was observed (Hernandez-Mendoza et al., 2009). These scientific observations elucidate the importance of temperature fluctuations in the production and accumulation of CLA. Lower temperature causes the bacterial cell membrane to lose its lipid bilayer fluidity, resulting in a phase transition in bacterial cells. The method of incorporating unsaturated fatty acids into the membrane is managed by thermal control. This phenomenon is observed in *E. coli* and other bacteria like *Enterococcus faecalis*, where bacteria alter their fatty acid composition by escalating cis-vaccenic acid content, consequently diminishing the incorporation of palmitic acid into the membrane phospholipid bilayer (Dong and Cronan, 2021). Lee et al. (2003) demonstrated efficient CLA production in washed cells of *Lb. reuteri*, where MRS media was supplemented with 0.45 g/L of LA at pH 9.5 with incubation at 37 °C for 1 h (Lee et al., 2003). Another investigation conducted by Jenkins and Courtney (2003) revealed that when *Lb. reuteri* ATCC 55739 was exposed to LA, the overall C18:2 content in their membranes increased from 18 to 50%. The study explained that roughly half of these C18:2 fatty acids consisted of LA, while the other half primarily comprised a single isomer of CLA. The enzyme responsible for the conversion of LA to CLA was LAI, which remained embedded in the cell membrane. This thermal regulatory mechanism enables microorganisms to adjust the fluidity of their cell membranes, optimizing their functionality across various growth temperatures (Jenkins and Courtney, 2003). Consequently, it is plausible that some of the LA present in the growth media were transformed by *Lb. reuteri* cells into isomers, with a portion being integrated into the cell membrane and another portion being released into the growth medium.

Again, the impact of changing pH was observed in CLA production. In one study, when *Lb. sakei* was cultured at 30 °C and with a pH change from 6.2 to 5.5, the bioconversion of LA to its conjugated isomers ceased, and the bacterial growth rate decreased significantly (Gorissen et al., 2011). In another study conducted on *P. pentosaceus*gs4, it was observed that the strain produced 149 μg/mL of CLA at pH 6.0, but the concentration decreased upon elevation of pH, producing 128 μg/mL of CLA isomer at pH 7.0. The amount further decreased to 63 μg/mL as the pH was increased to 9.0 after 48 h of incubation (Dubey et al., 2012). This study thus proves a direct correlation between CLA production and the pH of the media. Hence, the authors inferred that the optimum pH for CLA production ranges from 6.0 to 7.0, as increased pH (8.0–9.0) obstructs CLA formation, possibly by inhibiting the enzymatic activity of LAI and associated hydratase (Dubey et al., 2012). The degree of biohydrogenation might diminish if the integrity of the cell membrane is compromised, possibly due to exposure to an abnormal pH environment, as bacterial enzymes such as LAI are typically linked with cell membranes. In a previous study, Kepler and Tove (1967) documented that within a phosphate buffer system, the pH range for optimal isomerization of LA to CLA fell between 7.0 and 7.2. Decreased pH not only destabilized the isomerase enzyme but also impeded the micellar distribution of LA within the media (Kepler and Tove, 1967).

#### Incubation time

Dubey et al. (2012) experimentally showed that CLA formation by *P. pentosaceus* GS4 started at 6 h, with gradually increasing intensity, reaching a maximum at 48 h, followed by a drop in CLA levels. Similarly, another study showed a comparable observation using *Lb. fermentum* DDHI27 (Dahiya and Puniya, 2018). Wang et al. (2022) studied *Lb. casei* and found that the maximum CLA concentration was reached in the late stationary phase at around 72 h. CLA production starts in the log phase and spikes during the stationary phase. It is said that during these periods, cells are less susceptible to the toxic effect of CLA (Ogawa et al., 2005). These observations help in concluding that many factors such as culture condition, concentration of LA in the media, bacterial growth stages, and the isomerization ability of the enzyme LAI and its state of appearance greatly impact the produced CLA concentration.

### Application of LAI and other enzymes

#### Functional food production

LAI and associated enzymes find applications in the development of functional foods enriched with CLA. By leveraging the enzyme’s ability to convert LA into CLA, food scientists can enhance the nutritional profiles of food products. This includes the production of CLA-rich oils and fats, contributing to the development of health-promoting functional foods. Functional food is a type of food that is enriched with bioactive components or live microbes, which can impart certain health benefits (Guo, 2024). It is the best way to deliver micronutrients essential for human health (Nasrollahzadeh et al., 2023). These enzymes also serve as biocatalysts in the synthesis of CLA-rich oils and fats, contributing to the development of nutritional products with potential positive effects on human health. This application aligns with the growing interest in functional foods aimed at promoting wellbeing. Rumen bacteria metabolize polyunsaturated fatty acids, resulting in the formation of CLAs as an intermediary. CLA is a natural ingredient and occurs in numerous foods such as meat products, tissue fat, and dairy products (George and Ghosh, 2025). The range of total CLA concentration in dairy products or milk is 0.34 to 1.07% of total fat. The percentage of total fat that is composed of CLA in either raw or processed beef varies from 0.12 to 0.68%. Nowadays, it is believed that the average adult only consumes one-third to one-half of the CLA that has been demonstrated in animal tests to prevent cancer (Dhiman et al., 2005). Probiotic curd, yogurt, etc., could be prepared from various probiotics such as *P. pentosaceous* GS4 for CLA production and as potential functional food (Sukumar and Ghosh, 2013). Dairy products from ruminants are natural sources of CLAs; however, they do not contain much of them, so up-scaling of CLA concentration in these foods will raise the nutritional as well as therapeutic value of milk and meat products.

#### Biotechnological applications

LAI has become a hotspot for bacterial screening and manipulation. Many authors, such as Macouzet et al. (2010) and Gorissen et al. (2011), used LAI for screening the highest producer of CLA. Macouzet et al. (2010) also predicted the structure and molecular pattern of LAI-translated protein. Peng et al. (2007) attempted to isolate and purify the membrane-bound LAI protein from *C. sporogenes*. It was difficult to isolate the putative LAI protein, which confirmed its membrane association. In the case of *P. acnes*, the cytosolic presence of this protein makes it comparatively easier for isolation and characterization (Peng et al., 2007). Rosenberg and fellow researchers published their work in 2007 and became pioneers in showing genetically modified strains with the capacity to produce CLA with anti-proliferation properties (Rosberg-Cody et al., 2007). They used biotechnological means for the heterogeneous expression and overexpression of CLA trans-10, cis-12 isomers from *P. acne* to *Lactococcus lactis* and *E. coli*. On the other hand, Yang et al. (2017) utilized the cre-lox technique to study the working mechanism of different enzymes involved in the biotransformation of LA in *Lb. plantarum* ZS2058 for CLA production. In a different study conducted by Rosberg-Cody et al. (2011b), they demonstrated that the administration of a *Lactobacillus* strain modified to express the LAI enzyme pioneered the production of trans-10, cis-12 CLA, which could regulate the fatty acid composition in adipose tissue (Rosberg-Cody et al., 2011b), confirming the presence of the LAI gene. Hence, enzymes bioconverting LA have a pivotal role in the metabolism of unsaturated fatty acids and the production of CLA, which holds immense promise in various biotechnological applications. The unique catalytic abilities of these enzymes make them valuable tools in industries ranging from food production to pharmaceuticals.

#### Metabolic engineering for enhanced CLA production

Metabolic engineering strategies can be employed to enhance the expression of LAI and associated enzymes in microbial hosts, aiming for increased CLA production. This could involve optimizing the expression levels of enzymes, improving catalytic efficiency, and tailoring the host’s metabolic pathways to channel precursor flux toward CLA synthesis. Researchers, fascinated with the beneficial activity of CLA, are rigorously attempting to engineer high CLA production. Yang et al. (2017) created variants of *Lb. plantarum* ZS2058 through multiple genetic manipulations and concluded that three genes (MCRA, DH, and DC) are an integral part of the multiple-component, LAI system for the biotransformation of LA (Yang et al., 2017). Studies showed that bacterial lipolysis and proteolysis cause more CLA production. Lipolysis leads to an elevation in the level of CLA due to the accessibility of free fatty acid produced after the lipolysis of fat. Proteolysis, on the other hand, releases peptides and amino acids from protein, which act as a hydrogen donor in the CLA biohydrogenation reaction (Olivo et al., 2021; Moslemi et al., 2023).

CLA production was found to be strain-specific. The quantity and steps in bioconversion are different in strains of the same genus. *Bif. fibrisolvens* A38 has been reported to convert 40% LA to CLA, mostly cis-9, trans-11 CLA (Kim et al., 2003). In contrast, *Bif. fibrisolvens* TH1 showed more production and less accumulation of CLA as it transformed to trans-vaccenic acid (TVA) (Ross et al., 2010). Similarly, *Bif. fibrisolvens* MDT-5 produced and accumulated a considerable amount of CLA when CLA reductase activity was halted (Fukuda et al., 2006).

## Future perspectives and challenges

The future trajectory of LAI and associated enzyme research holds exciting prospects with potential breakthroughs in understanding its intricate mechanisms, expanding its applications, and unraveling novel roles in cellular physiology. Despite substantial studies on individual CLA-producing enzymes, there is still a lack of a concrete understanding of their functional interplay, mechanistic convergence/divergence, and evolutionary origin. Additionally, the precise roles of MCRA proteins and hydratases in CLA biosynthesis, their overlap with canonical LAI activity, and the extent to which these enzymes operate within unified or modular pathways are still under active investigation. Furthermore, discrepancies between sequence homology and functional output across different enzyme families highlight a critical limitation in predicting enzymatic roles based solely on genomic information. These gaps demonstrate the necessity for an integrative perspective that connects enzyme structure, catalytic function, and metabolic context.

Beyond this, the regulatory architecture governing CLA biosynthesis remains poorly resolved. According to scientific literature, the expression of the CLA operon is regulated by transcriptional regulators such as LysR-type transcriptional regulators (LTTR) and environmental cues, including substrate availability, oxygen levels, and physicochemical conditions. However, there is still an inadequate systems-level understanding of the mechanism of the regulatory network, which coordinates enzyme activity and metabolic flux toward cellular CLA manufacturing. This review provides extensive insight into CLA and the enzymes responsible for its production in microorganisms. Enzymes converting LA to CLA are classified, and there has been an extensive study of LAI from GI. However, the GII class needs more exploration and extensive studies.

The available records suggest that the ways to trace the presence of LAI in an organism are by assessing the level of CLA through spectrophotometric, chromatographic, and genetic approaches. One of the main difficulties with a genomic approach is that the predicted enzyme function is based on genetic homology with other annotated proteins, without considering the fact that different members of the same family of proteins may have different functional roles (Engelhardt et al., 2009).

Potential genes in CLA generation need to be identified. After identifying a potential gene, the subsequent step should be to confirm its catalytic role. More research should be performed to clearly understand the biohydrogenation process. However, experiments following insertional mutagenesis revealed that none of the integration mutants were able to present expected results *(*Macouzet et al., 2010). This could be because the functional products of the studied genes are essential for the bacteria to survive. If the conjugation process serves as a detoxification mechanism, these genes might not be directly involved in simple LA isomerization but could play a role in a more intricate reaction pathway of essential genes. Further investigations aimed at confirming this hypothesis could involve antisense RNA to reduce both transcript and protein levels without completely disabling the gene. This approach should be complemented with transcriptomic, proteomic, and lipidomic analyses.

Finally, multi-component enzymatic systems suggest several challenges that need to be addressed, specifically: (i) obtaining strong evidence to determine whether individual proteins or a multi-enzymatic complex are responsible for LA conversion, and (ii) elucidating their kinetic parameters. This will enable us to understand why certain strains exhibit a higher capacity to convert LA to CLA than others. However, estimation of CLA can be a costly procedure, so research can also be focused on developing an affordable alternative.

## Conclusion

Due to its immense benefit on human health, CLA has gained significant attention. CLA is anti-inflammatory, anti-cancer, anti-diabetic, helps in developing bone health, managing body weight, preventing atherosclerosis, and also boosts immunity. Hence, the incorporation of CLA as a functional food in the diet is a good way of providing nutrition to the human body and preventing lifestyle diseases. However, the application and usage of CLA in humans need more research and extensive clinical trials.

The ubiquitous occurrence of CLA-producing biohydrogenating enzymes such as LAI, OleH, MCRA, and fatty acid hydratases, from bacteria to fungi and beyond, underscores their ecological relevance, evolutionary adaptability, and diversification of substrate-enzyme interactions. The multifaceted exploration of these enzymes reveals their importance in cellular fatty acid metabolism. Collectively, the ecological distribution and evolutionary diversification of CLA-producing enzymes indicate that microbial biohydrogenation pathways evolved as adaptive metabolic strategies for survival, membrane regulation, and detoxification in lipid-rich environments.

The elucidation of the molecular structure, catalytic mechanisms, and substrate specificity of these enzymes provides a stable foundation for understanding the mechanism of CLA biosynthesis. However, despite substantial advances in understanding their structure, catalytic mechanisms, and regulatory pathways, several aspects of enzyme cooperation, functional divergence, and metabolic regulation remain unresolved. Future studies integrating molecular biology, structural bioinformatics, transcriptomics, and metabolic engineering will be essential for improving CLA biosynthesis and exploiting these enzymes for industrial and therapeutic applications.

## Glossary

A33: alanine 33
ATCC: American Type Culture Collection
E57: glutamic acid 57
G31: glycine
PDB: protein data bank
CLA: conjugated linoleic acid
LA: linoleic acid
OA: Oleic acid
SA: stearic acid
RA: ricinoleic acid
TVA: trans-vaccenic acid
LAI: linoleate isomerase
MCRA: myosin cross-reactive antigen protein
PAI: polyunsaturated fatty acid isomerase
FA-HY1/2: fatty acid hydratase 1/2
OleH: ohyA oleate hydratase
SCD1: Δ9 desaturase
LNA: linolenic acid
CLNA: conjugated linolenic acid
antisense RNA: ribonucleic acid
MCT: medium chain triglycerides
UCP-1: uncoupling protein of respiratory chain-1
CLA-R: conjugated linoleic acid reductase
CLA-HY: CLA-oleate hydratase
CLA-DH: CLA-short chain dehydrogenase
CLA-DC: CLA-acetoacetate decarboxylase
CLA-ER: CLA-enone reductase
CLA-FFA: CLA-free fatty acid
MMP-2/9: matrix metalloprotease
Fab1: enoyl-acyl carrier protein reductase
gdhA: glutamate dehydrogenase
NADP: nicotinamide adenine dinucleotide phosphate
LTTR: LysR-type transcriptional regulator
FAD: flavin adenine dinucleotide
13-LAH: linoleate 13-hydratase
10-LAH: linoleate 10-hydratase
13-HOE: 13-hydroxy-cis-9-octadecanoic acid
10-HOE: 10-hydroxy-cis-12-octadecanoic acid
10-HOA: 10-hydroxy octadecanoic acid
MRS: de Mann Rogosa Sharpe
LAB: lactic acid bacteria
MHC II: Major Histocompatibility Complex II
NC-IUBMB: The Nomenclature Committee of the International Union of Biochemistry and Molecular Biology
IBD: inflammatory bowel disease
PPAR: peroxisome proliferator activated receptor.
